# Effect and mechanism of Emodin on mouse myeloid-derived suppressor cells

**DOI:** 10.3389/fphar.2026.1840325

**Published:** 2026-09-14

**Authors:** Yating Shu, Li Ma, Weiling Xiao, Xingyue Li, Xiaotian Qi, Meiyu Peng

**Affiliations:** 1 Key Laboratory of Immune Microenvironment and Inflammatory Disease Research in Universities of Shandong Province, School of Basic Medical Sciences, Shandong Second Medical University, Weifang, China; 2 Department of Nephrology, Affiliated Hospital of Shandong Second Medical University, Weifang, China; 3 Digestive Disease Diagnosis and Treatment Center, Affiliated Hospital of Shandong Second Medical University, Weifang, China

**Keywords:** AKT/NF-κB/STAT3/STAT1, Emodin, myeloid-derived suppressor cells, pancreatic cancer, tumor immune microenvironment

## Abstract

Emodin, a natural anthraquinone compound, exhibits anti-inflammatory, antioxidant, and broad-spectrum anti-tumor activities. Studies have shown that Emodin can inhibit the activation and functions of various immune cells, thereby being recognized as an important immunosuppressive agent. In this study, we conducted *in vitro* experiments to examine the effects of Emodin on the apoptosis, differentiation, and immunosuppressive functions of myeloid-derived suppressor cells (MDSCs), as well as its impacts on the migration, proliferation, and viability of mouse pancreatic cancer H7 cells. Furthermore, we established a mouse orthotopic pancreatic cancer model to evaluate Emodin’s anti-pancreatic cancer effects *in vivo* and its modulation of immune cell subpopulations. The results revealed that Emodin exerts anti-pancreatic cancer effects through direct inhibition of tumor cells and regulation of MDSCs, although its modulation of the immune microenvironment exhibits concentration-dependent and subset-specific characteristics. These findings suggest that combination therapy and dose optimization are crucial directions for its clinical translation.

## 1 Introduction

Pancreatic cancer presents an insidious onset, rapid progression and poor clinical prognosis, with a 5-year survival rate of only 13%. Conventional interventions, including surgical resection, radiotherapy and chemotherapy, have reached a therapeutic plateau in improving the long-term survival of patients with pancreatic cancer, leaving limited room for further clinical breakthroughs (Halbrook et al., 2023). In recent years, immunotherapy has emerged as a transformative therapeutic modality and achieved remarkable outcomes in multiple solid tumors, including melanoma and lung cancer. Nevertheless, its clinical efficacy against pancreatic cancer remains suboptimal (Mukherji et al., 2022). Such unsatisfactory therapeutic responses stem principally from the profoundly immunosuppressive tumor immune microenvironment of pancreatic cancer, which is typified by impaired infiltration and severe functional suppression of tumor antigen-specific CD8^+^ T cells, as well as abnormal accumulation of immunosuppressive cells (Silva et al., 2024). In this context, exploring novel therapeutic strategies to remodel the pancreatic tumor immune microenvironment and convert “cold” tumors into “hot” ones is critically important to overcome the current therapeutic bottleneck in pancreatic cancer treatment.

Myeloid-derived suppressor cells (MDSCs) constitute a pivotal component of the immunosuppressive tumor microenvironment and exert potent immunoinhibitory functions. MDSCs dampen the activation and effector functions of CD8^+^ T cells by secreting multiple immunosuppressive mediators, including arginase-1 (Arg-1), reactive oxygen species (ROS), and inducible nitric oxide synthase (iNOS) (Tong et al., 2025). Furthermore, MDSCs facilitate the induction and expansion of regulatory T cells (Treg), thereby collaboratively amplifying T cell immunosuppression (Fujimura et al., 2012). Accordingly, therapeutic strategies targeting MDSCs have become a major research hotspot in oncology. Current MDSCs-targeted interventions primarily focus on three dimensions: (1) reducing MDSCs accumulation (Savardekar et al., 2025); (2) abrogating the immunosuppressive activity of MDSCs (Xun et al., 2024); (3) blocking MDSCs migration and recruitment (Sun et al., 2023). Such strategies are expected to alleviate tumor immunosuppression in pancreatic cancer and ultimately improve antitumor therapeutic efficacy.

Emodin is a natural anthraquinone compound extracted from multiple medicinal plants, including Rheum palmatum and Polygonum multiflorum, and possesses potent anti-inflammatory, antioxidant, and broad-spectrum anti-tumor properties (Dong et al., 2016). Accumulating studies have demonstrated that Emodin suppresses pancreatic cancer progression by facilitating pancreatic cancer cell apoptosis (Liu et al., 2012), inhibiting tumor invasion (Li et al., 2018), and abrogating tumor angiogenesis (Lin et al., 2012). In addition to its direct tumoricidal effects, the immunomodulatory capacity of Emodin has also attracted widespread research attention. Existing evidence indicates that Emodin inhibits the secretion of T helper 1 (Th1) and Th17 cell-related cytokines, while upregulating the expression of interleukin-4 (IL-4) and IL-10. This effect remodels the Th1/Th2 and Th17/Treg immune balance and suppresses Con A-induced proliferation and autoimmune responses in murine spleen cells (Sharma and Tiku, 2016). Nevertheless, comprehensive and systematic studies regarding the anti-tumor mechanisms of Emodin via remodeling the tumor immune microenvironment, especially through targeting MDSCs, remain insufficient. Accordingly, the present study focuses on the regulatory effects of Emodin on MDSCs apoptosis, differentiation, and immunosuppressive functions, aiming to explore its therapeutic efficacy against pancreatic cancer and its modulatory role in the tumor immune microenvironment. This work provides reliable experimental evidence to support the clinical translation of Emodin for pancreatic cancer treatment.

## 2 Materials and methods

### 2.1 Experimental animals

Six-to eight-week-old female specific pathogen-free (SPF) C57BL/6J mice were purchased from Jinan Pengyue Experimental Animal Breeding Co., Ltd. All animals were maintained in an individually ventilated cage (IVC) system at the Immunology Laboratory of Shandong Second Medical University. Following 7 days of acclimation, all mice were used for subsequent formal experiments. All animal experimental procedures were approved by the Animal Ethics Committee of Shandong Second Medical University (Approval No.: 2024SDL307).

### 2.2 Establishment of the tumor model

H7 cells were cultured in Dulbecco’s Modified Eagle Medium (DMEM, Gibco, USA) supplemented with 10% fetal bovine serum (Gibco, USA) and 100 U/mL penicillin-streptomycin solution (Solarbio, China) at 37 °C in a humidified incubator with 5% CO_2_. For *in vivo* pancreatic implantation, cultured H7 cells were harvested, and 1 × 10^6^ cells were resuspended in 50 μL of sterile phosphate-buffered saline (PBS) for subsequent injection. Prior to surgery, mice were anesthetized, and the abdominal skin was disinfected with povidone-iodine. A 1 cm longitudinal incision was made on the upper left abdomen. The pancreas and spleen were gently elevated to expose the pancreatic tail and fully visualize the pancreatic head. The prepared cell suspension was slowly injected from the pancreatic tail toward the pancreatic head using a 27-gauge needle. After injection, the pancreatic and splenic tissues were repositioned, and the peritoneum and skin were sequentially sutured for wound closure.

### 2.3 Acquisition of bone marrow cells

Mice were humanely euthanized by CO_2_ asphyxiation and surface-disinfected with 75% ethanol. All tissue isolation procedures were performed under strict aseptic conditions. Briefly, the skin of the hind limbs was removed, and intact hind limb bones were harvested by disarticulation at the ankle and hip joints. Soft muscle and connective tissues adhering to the bone surface were thoroughly cleared, and the epiphyseal ends were cut to expose the intramedullary cavity. The marrow cavity was repeatedly irrigated with sterile PBS using 1-mL syringes, and the flushed bone marrow cells (BMC) suspension was collected for subsequent experiments.

### 2.4 Magnetic bead sorting of MDSCs from tumor-bearing mice and purity assessment

Single-cell suspensions were prepared from the cardiac blood, spleen, and bone marrow of tumor-bearing mice. After red blood cell lysis, cells were incubated with Ly6C and Ly6G magnetic beads (BD Biosciences, USA) at 4 °C for 15 min. Twenty volumes of 1× magnetic bead sorting buffer were applied to remove unbound cells, and the supernatant was discarded after centrifugation. The cell pellet was resuspended in 2 mL of 1× sorting buffer and placed on a magnetic rack, followed by supernatant removal. MDSCs purity was assessed by flow cytometry. Briefly, cell aliquots were preincubated with CD16/32 antibody (Biolegend, USA) at 4 °C to block Fc receptors for 15 min, and then stained with CD11b-FITC, Ly6C-APC, and Ly6G-PE antibodies (Biolegend, USA). Samples were analyzed using a BD FACSVerse flow cytometer, and the sorted MDSCs purity exceeded 90%.

### 2.5 Detection of MDSCs apoptosis

Sorted MDSCs derived from tumor-bearing mice were adjusted to a concentration of 1 × 10^6^ cells/mL and seeded in 24-well plates at a final volume of 1 mL per well. Each well was supplemented with 1 μL of 20 μg/mL recombinant mouse GM-CSF (Sigma-Aldrich, USA) and different concentrations of Emodin (0, 20, and 40 μmol/L; Sigma-Aldrich, USA), followed by 24 h of incubation. After treatment, cells were harvested and stained with 5 μL of FITC Annexin V and 5 μL of 7-AAD (BioLegend, USA). After 30 min of incubation at room temperature in the dark, cell apoptosis was analyzed using a BD FACSVerse flow cytometer.

### 2.6 Detection of MDSCs differentiation

Bone marrow cells were resuspended and adjusted to a concentration of 1 × 10^6^ cells/mL in RPMI 1640 complete medium supplemented with 10% fetal bovine serum, and seeded in 24-well plates at a final volume of 1 mL per well. Each well was supplemented with 1 μL of 20 μg/mL recombinant mouse GM-CSF (Sigma-Aldrich, USA) and different concentrations of Emodin (0, 20, and 40 μmol/L; Sigma-Aldrich, USA), followed by 4 days of incubation. During culture, half of the medium was refreshed every 2 days. After incubation, cells were harvested and counted, and then stained with anti-mouse CD11b-FITC, Ly6C-APC, and Ly6G-PE antibodies (all Biolegend, USA). The proportions of total MDSCs, M-MDSCs, and PMN-MDSCs were quantified using a BD FACSVerse flow cytometer.

### 2.7 Detection of Arg-1 and ROS levels

MDSCs were isolated from tumor-bearing mice via magnetic bead sorting and treated with culture medium containing different concentrations of Emodin (0, 20, and 40 μmol/L; Sigma-Aldrich, USA) for 3 h. After treatment, cells were harvested to detect intracellular ROS levels using a ROS detection kit (MedChemExpress, USA). For intracellular Arg-1 staining, cells were first surface-stained with mouse FITC-CD11b, Ly6G-PE, and Ly6C-BV421 antibodies (all Biolegend, USA) at 4 °C for 30 min, followed by fixation and permeabilization. Cells were then incubated with Arg-1-APC antibody (Invitrogen, USA) at 4 °C for 30 min, followed by data acquisition on a BD FACSVerse flow cytometer.

### 2.8 Real-time quantitative PCR

MDSCs were isolated from tumor-bearing mice by magnetic bead sorting, seeded at a density of 2×10^7^ cells per 6-cm dish with a final volume of 6 mL per dish. Each dish was supplemented with 6 μL of 20 μg/mL recombinant mouse GM-CSF (Sigma-Aldrich, USA) and different concentrations of Emodin (0, 20, and 40 μmol/L; Sigma-Aldrich, USA), followed by 24 h of incubation. After incubation, cells were harvested, and total RNA was extracted using TRIzol reagent (Invitrogen, USA). First-strand cDNA was synthesized using a reverse transcription kit (Thermo Fisher Scientific, USA) in accordance with the manufacturer’s instructions. Glyceraldehyde-3-phosphate dehydrogenase (GAPDH) was used as the internal reference gene for qPCR detection. The primer sequences used for qPCR were as follows:

Arg-1 (F) 5′-TGT​CCC​TAA​TGA​CAG​CTC​CTT-3′,

(R) 5′-GCA​TCC​ACC​CAA​ATG​ACA​CAT-3’; GAPDH (F) 5′-TGT​CTC​CTG​CGA​CTT​CAA​CA-3′,

(R) 5 ‘-GGT​GGT​CCA​GGG​TTT​CTT​ACT-3’.

All qPCR experiments were performed on a Roche LightCycler 480 real-time quantitative PCR system. Relative gene expression levels were calculated using the 2^−*ΔΔCt*^ method, followed by subsequent statistical analysis.

### 2.9 MDSCs inhibition of T cell proliferation detection

MDSCs were isolated from tumor-bearing mice by magnetic bead sorting and adjusted to a concentration of 1×10^6^ cells/mL. Spleen cells isolated from normal mice were lysed with red blood cell lysis buffer (Solarbio, China), labeled with CFSE (Thermo Fisher Scientific, USA), and resuspended at 1 × 10^6^ cells/mL. MDSCs and CFSE-labeled splenocytes were co-cultured in plates at a cell ratio of 2:1. The experimental groups were set as follows: ① Con A+ splenocytes group; ② Con A+ splenocytes + MDSCs group; ③ Con A+ splenocytes + different concentrations of Emodin group (0, 20, 40 μmol/L); ④ Con A+ splenocytes + MDSCs + different concentrations of Emodin group (0, 20, 40 μmol/L). Con A (Sigma-Aldrich, USA) was added at a final concentration of 2 μg/mL. After 4 days of co-culture, T cell proliferation was detected using a BD FACSVerse flow cytometer.

### 2.10 WB pathway detection

MDSCs were isolated from tumor-bearing mice via magnetic bead sorting and adjusted to a concentration of 2×10^7^ cells/mL. Cells were pretreated with different concentrations of Emodin (0, 20, and 40 μmol/L; Sigma-Aldrich, USA) for 6 h, followed by stimulation with 20 μg/mL recombinant mouse GM-CSF (Sigma-Aldrich, USA) for 30 min. Subsequently, total cellular protein was extracted using RIPA lysis buffer (Solarbio, China), and protein concentrations were quantified using the BCA assay to ensure consistent protein loading across all groups. Protein samples were denatured by boiling at 100 °C for 10 min. A total of 30 μg of protein per sample was subjected to SDS-PAGE electrophoresis. Electrophoresis was performed at a constant voltage of 80 V for the stacking gel and 120 V for the separating gel until the protein bands reached the bottom of the gel. Separated proteins were electrotransferred onto PVDF membranes at a constant current of 210 mA for 75 min. After transfer, the membranes were blocked with 5% BSA solution at room temperature for 1.5 h on a shaking platform. Following blocking, the membranes were incubated with primary antibodies at 4 °C overnight. After thorough washing, the membranes were incubated with corresponding secondary antibodies at room temperature for 1 h. Protein bands were visualized by chemiluminescence imaging, and ImageJ software was applied for grayscale analysis to determine the relative expression levels of target proteins.

### 2.11 Cell scratch assay

H7 cells were seeded in 6-well plates and cultured in complete DMEM medium. When cells confluence reached approximately 70%, the medium was replaced with serum-free medium for overnight culture to obtain a uniform cell monolayer. A linear scratch was generated on the cell monolayer using a 10 μL pipette tip. The cells were then cultured in DMEM containing 2% FBS and different concentrations of Emodin (0, 20, and 40 μmol/L; Sigma-Aldrich, USA). The scratch wounds were imaged under a microscope at ×20 magnification at 0 h and 24 h. All experiments were performed in triplicate. The scratch width was measured using ImageJ software for statistical analysis.

### 2.12 Cell proliferation assay

H7 cells were starved overnight and stained with CFSE for proliferation detection. Briefly, cells were digested and washed, resuspended in DMEM medium containing 5 μM CFSE (Thermo, USA), and incubated at 37 °C for 10 min in the dark. Complete medium was added to terminate the staining reaction. CFSE-labeled cells were treated with different concentrations of Emodin (0, 20, and 40 μmol/L; Sigma-Aldrich, USA) for 24 h. After digestion and washing, cell proliferation was analyzed using a BD FACSVerse flow cytometer.

### 2.13 Cell viability assay

H7 cells were seeded in 96-well plates and cultured overnight until fully adherent. The cells were then treated with different concentrations of Emodin (0, 20, and 40 μmol/L; Sigma-Aldrich, USA) for 24 h. Afterwards, 10 μL of CCK-8 reagent (Solarbio, China) was added to each well, followed by incubation at 37 °C for 1 h in the dark. The absorbance was measured at 450 nm with a microplate reader, and the relative cell viability was calculated.

### 2.14 Toxicity detection of Emodin in mice

Six-week-old healthy C57BL/6 J mice were randomly assigned to vehicle control, 40 mg/kg Emodin (Sigma-Aldrich, USA) and 80 mg/kg Emodin (Sigma-Aldrich, USA) groups. Mice were intraperitoneally injected three times per week for two consecutive weeks before euthanasia, and cardiac blood as well as liver and kidney tissues were collected.

### 2.15 Detection of serum alanine transaminase (ALT) and aspartate transaminase (AST)

Serum ALT/AST activity was quantified using a microplate colorimetric kit (Nanjing Jiancheng, China). 20 μL pre-warmed substrate solution and 20 μL chromogenic reagent were mixed with 5 μL sample (or blank control) and incubated at 37 °C for 50 min. The reaction was terminated by adding 200 μL stop solution, followed by a 15-min room-temperature equilibration. Absorbance was read at 505 nm, and absolute OD values were calculated by subtracting control well absorbance. ALT/AST activity (U/L) was determined via standard curve fitting and multiplied by a conversion factor (0.482) and sample dilution fold.

### 2.16 Detection of blood urea nitrogen (BUN)

BUN was determined using a urea nitrogen colorimetric assay kit based on the urease method (Nanjing Jiancheng, China). Briefly, 0.02 mL of double-distilled water, 10 mmol/L urea nitrogen standard working solution, and test sample were added to blank, standard, and test tubes, respectively, followed by 0.25 mL urease working solution into each tube. The mixtures were incubated in a water bath at 37 °C for 10 min. Subsequently, 1 mL chromogenic reagent and 1 mL buffer solution were sequentially supplemented, and the solutions were mixed thoroughly before a light-shielded water bath incubation at 37 °C for another 10 min. The absorbance was measured at 640 nm. The BUN concentration (mmol/L) was calculated according to the following formula: Calculate using the formula: BUN (mmol/L) = (Sample A–Blank A)/(Standard A–Blank A) × 10 × dilution factor.

### 2.17 Detection of serum creatinine (CRE)

Serum CRE levels were quantified using a commercial creatinine detection kit (Nanjing Jiancheng, China) based on the creatine kinase enzymatic method in 96-well microplates. Briefly, 6 μL of test sample, creatinine standard or double-distilled water (blank control) was loaded into each well, followed by the sequential addition of 6 μL Reagent 3 and 180 μL Enzyme Solution A. The mixture was incubated at 37 °C for 5 min, and the absorbance value A_1_ was recorded at 546 nm. Subsequently, 60 μL Enzyme Solution B was supplemented, and the plate underwent another 5-min incubation at 37 °C before measuring absorbance A_2_ at the same wavelength. After blank background correction, the adjusted absorbance difference ΔA was calculated as ΔA = A_2_ − (186/246) × A_1_. The final creatinine concentration was computed by substituting ΔA into the kit-provided calculation formula and further multiplying by the sample dilution factor.

### 2.18 Hematoxylin-eosin (HE) staining of liver and kidney tissues

Liver and kidney tissues were fixed in 4% PFA for 24 h, rinsed with running water, dehydrated, cleared and embedded in paraffin. 4 μm-thick sections were prepared, dewaxed and rehydrated before hematoxylin-eosin staining, followed by secondary dehydration, clearing and mounting. Five random high-power fields were captured from each section for lesion observation.

### 2.19 Detection of Emodin’s effect on immune cell numbers *In vivo*

A mouse orthotopic pancreatic cancer model was successfully established. After a 7-day recovery period, the mice were randomly divided into three groups (n = 6). Mice in the experimental groups received intraperitoneal injection of Emodin (40 and 80 mg/kg; Sigma-Aldrich, USA), three times per week for two consecutive weeks. Mice in the control group were intraperitoneally administered with an equal volume of vehicle consisting of 5% DMSO (Solarbio, China), 40% PEG300 (MedChemExpress, USA), 5% Tween-80 (MedChemExpress, USA), and 50% normal saline. After treatment, all mice were euthanized. Body weight was recorded, and spleen and tumor tissues were isolated. Tissues were ground and filtered through a 40 μm cell strainer to prepare single-cell suspensions, followed by red blood cell lysis using red blood cell lysis buffer (Solarbio, China). The cell concentration was adjusted to 1 × 10^6^ cells/mL, and 100 μL of cell suspension was collected for flow cytometric staining. The immunocyte subgroup staining procedures were described as follows: (1) T cells: CD45-BV421, CD3-Percp, CD4-APC-H7, and CD8-APC (all BD Biosciences, USA). (2) MDSCs: CD45-BV421 (BD Biosciences, USA), CD11b-FITC, Ly6C-APC, and Ly6G-PE (all Biolegend, USA). All cell samples were incubated at 4 °C for 30 min before flow cytometry detection. (3) Treg: cells were first surface-stained with CD4-APC-H7 (BD Biosciences, USA) and CD25-APC (Invitrogen, USA) at 4 °C for 30 min. After fixation and permeabilization with 1 mL fixing/permeabilization solution (Invitrogen, USA) for 30 min and washing with 2 mL 1× permeabilization buffer (Invitrogen, USA), cells were incubated with Foxp3-PE (Invitrogen, USA) at 4 °C for another 30 min and finally detected using a BD FACSVerse flow cytometer.

### 2.20 Statistical analysis

All experimental data are expressed as mean ± standard deviation (SD). Intergroup differences were assessed via one-way analysis of variance (ANOVA). GraphPad Prism 9.5 was used for statistical analyses, *p*-value <0.05 was considered statistically significant.

## 3 Results

### 3.1 The dosage of Emodin *in vitro* and *in vivo*

To screen optimal Emodin concentrations for subsequent MDSCs functional assays, we first generated a dose–response apoptosis curve and determined the half-maximal inhibitory concentration (IC_50_) of Emodin. As shown in Supplementary Figure S1, data from five serially diluted concentration groups were fitted to a four-parameter logistic model using the Conduct Science IC_50_ Calculator based on the unweighted least squares method, Consistent with previous *in vitro* studies of Emodin against pancreatic cancer, most experiments used 20 μmol/L and 40 μmol/L as intervention concentrations (Li et al., 2018), these two concentrations were selected for all subsequent *in vitro* functional assays in the present study.

Previous studies adopted intraperitoneal Emodin doses of 40 mg/kg and 80 mg/kg in mouse pancreatic cancer models (Lin et al., 2015). Pharmacological evidence has also confirmed that oral administration of Emodin at 20, 40, and 80 mg/kg is well tolerated for continuous treatment without overt toxicity for up to 12 weeks in both male and female mice (Sougian et al., 2021). To further validate the *in vivo* safety of the applied dosages, six-week-old healthy C57BL/6 J mice were intraperitoneally injected with 40 mg/kg or 80 mg/kg Emodin three times weekly for two consecutive weeks. As shown in Supplementary Figures 2AB, no prominent pathological lesions, including inflammatory infiltration and cellular necrosis, were observed in the liver and kidney tissues of Emodin-treated mice relative to vehicle controls. Consistent with the histological results, serum biochemical analysis (Supplementary Figure S2C) showed no significant differences in ALT, AST, CRE, BUN levels across the three groups. The corresponding values were as follows: ALT (10.08 ± 1.96 U/L vs. 11.47 ± 2.70 U/L vs. 10.08 ± 3.30 U/L, *P* > 0.05), AST (11.76 ± 3.47 U/L vs. 13.64 ± 2.71 U/L vs. 13.14 ± 3.72 U/L, *P* > 0.05), CRE (23.74 ± 1.99 μmol/L vs. 24.44 ± 1.16 μmol/L vs. 22.70 ± 1.36 μmol/L, *P* > 0.05), and BUN (11.20 ± 0.96 mmol/L vs. 11.85 ± 0.60 mmol/L vs. 13.44 ± 2.17 mmol/L, *P* > 0.05). Collectively, these results demonstrate that the two Emodin dosages used in this study do not induce apparent hepatic or renal damage, supporting the satisfactory *in vivo* safety profile of this treatment regimen.

Apoptosis rates under five Emodin concentrations were fitted with a 4  PL logistic model (unweighted least squares, R^2^ = 0.9990). The IC_50_ value was calculated as 39.27 μmol/L with a Hill slope of 1.941.

### 3.2 Emodin induces apoptosis in MDSCs

Accumulating evidence demonstrates that Emodin exerts suppressive effects on multiple immune cell subsets, but its impact on MDSCs apoptosis has not yet been reported. Therefore, this study used the Annexin-V/7-AAD double staining method to assess the apoptotic effects of Emodin on MDSCs. As shown in Figure 1, compared to the untreated group, the overall apoptosis rate of MDSCs in the 20 μmol/L and 40 μmol/L Emodin treatment groups (29.47% ± 10.72% vs. 37.28% ± 10.14% vs. 59.34% ± 14.66%, *P* < 0.01), early apoptosis rate (18.64% ± 8.01% vs. 24.64% ± 8.35% vs. 35.02% ± 7.64%, *P* < 0.05), and late apoptosis rate (10.83% ± 2.84% vs. 12.64% ± 1.99% vs. 24.32% ± 7.57%, *P* < 0.01) were markedly elevated. These results suggest that Emodin promotes apoptosis in MDSCs, and this effect intensifies with increasing Emodin concentration.

**FIGURE 1:**
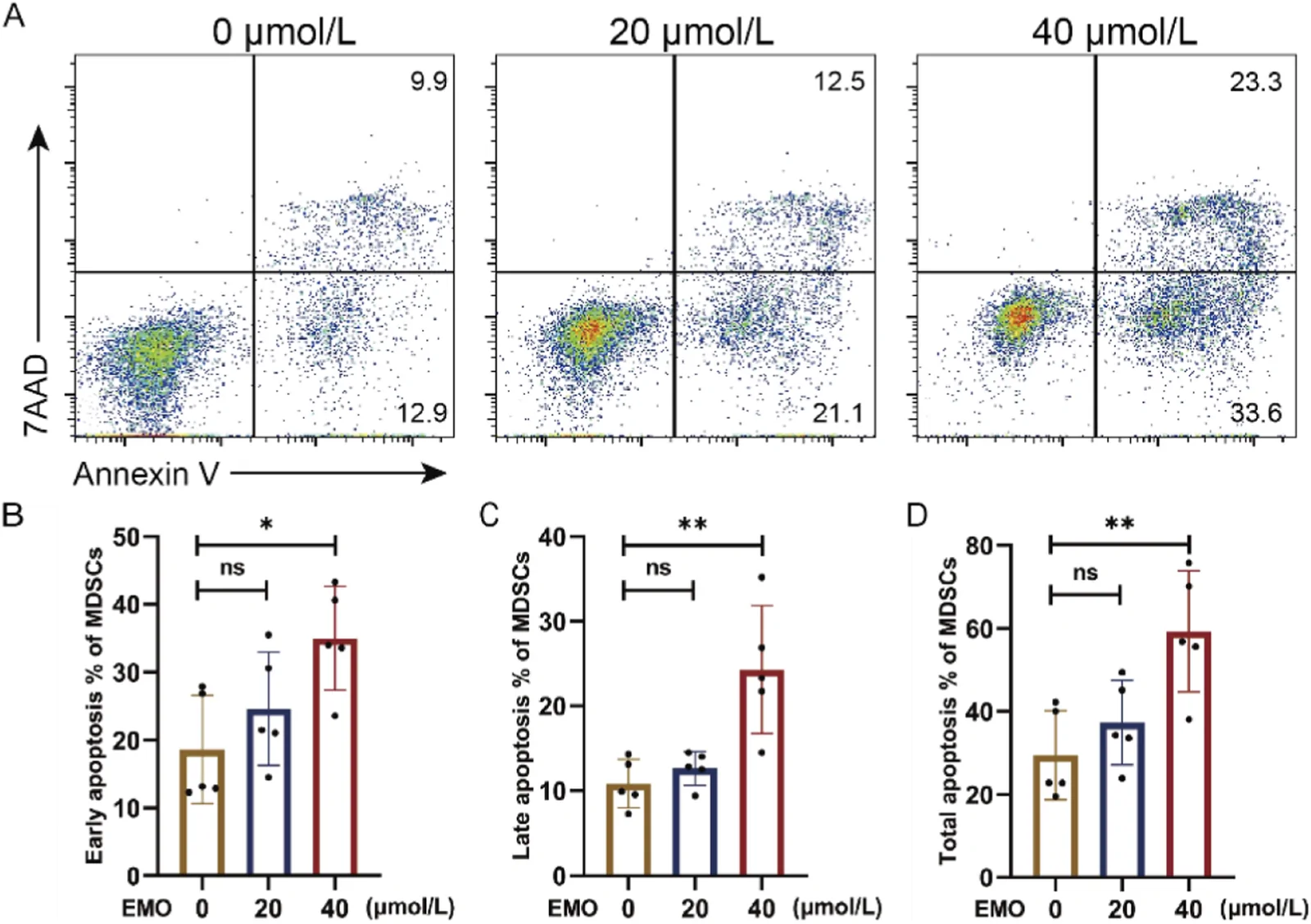
Effects of Emodin on MDSCs apoptosis. **(A)** Representative flow plots of MDSCs apoptosis. **(B)** Percentage of early apoptosis in MDSCs. **(C)** Percentage of late apoptosis in MDSCs. **(D)** Total apoptosis percentage in MDSCs (n = 5).

### 3.3 Effects of Emodin on MDSCs differentiation

Mouse MDSCs consist of two subtypes: PMN-MDSCs (CD11b^+^ Ly6G^+^ Ly6C^low^) and M-MDSCs (CD11b^+^ Ly6G^−^ Ly6C^high^), both of which exert immunosuppressive effects through distinct mechanisms. To explore the impact of Emodin on MDSCs differentiation, this study used MDSCs induced *in vitro* from normal mouse BMC. During GM-CSF-induced differentiation of normal mouse BMC into MDSCs, cells were cultured with various concentrations of Emodin for four consecutive days. FCM analysis revealed significant shifts in the proportions of MDSCs subtypes following Emodin treatment. As shown in Figure 2B, compared with the untreated group, the proportion of PMN-MDSCs progressively increased in the 20 μmol/L and 40 μmol/L Emodin treatment groups (32.48% ± 2.37% vs. 38.42% ± 4.26% vs. 43.08% ± 4.98%, *P* < 0.01), while the proportion of M-MDSCs decreased correspondingly (59.32% ± 2.76% vs. 54.76% ± 4.31% vs. 51.62% ± 4.52%, *P* < 0.05). However, the total proportion of MDSCs showed no significant change (91.80% ± 1.40% vs. 93.42% ± 1.60% vs. 94.70% ± 2.33%, *P* > 0.05).

**FIGURE 2:**
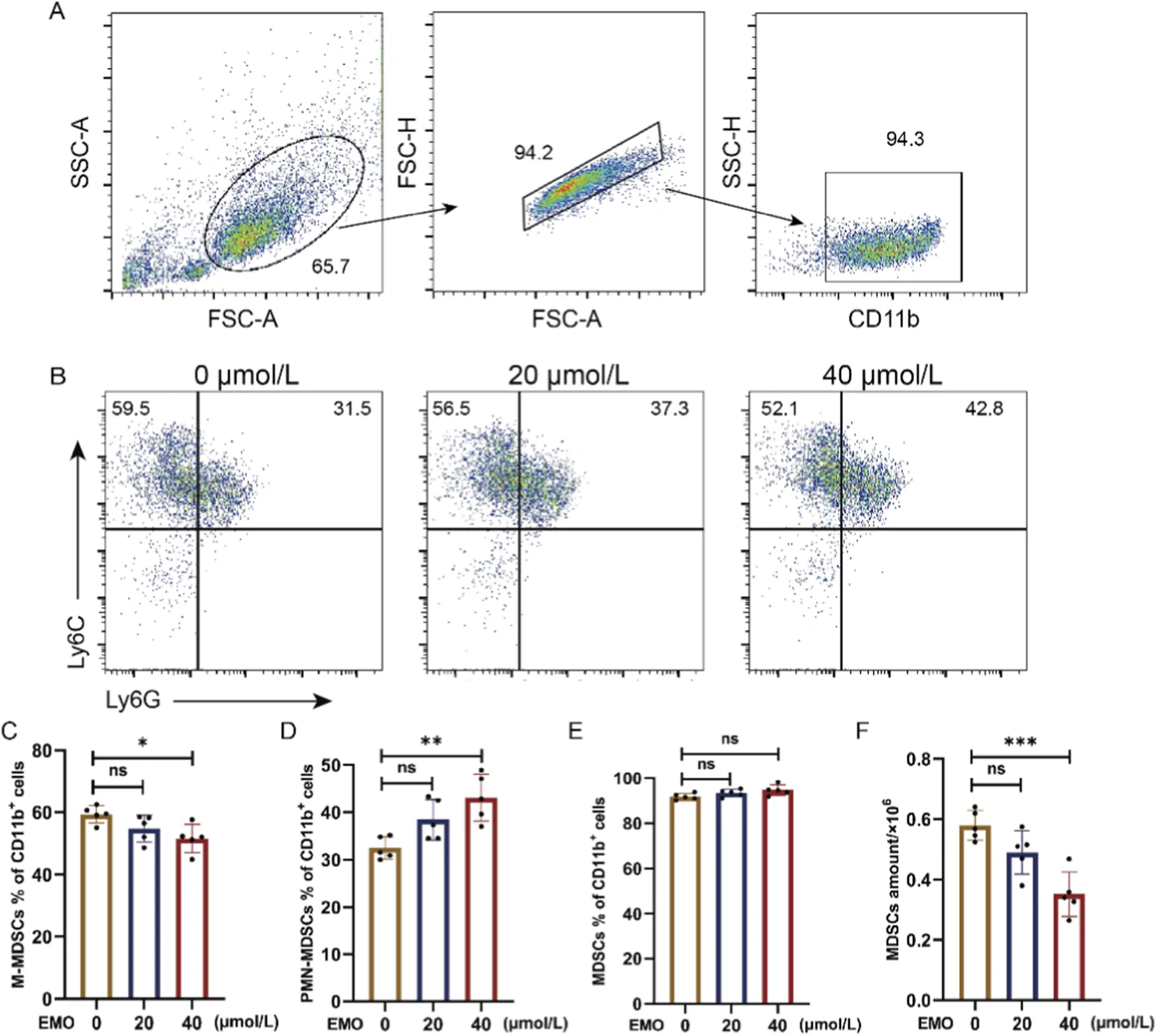
Effects of Emodin on MDSCs differentiation. **(A)** MDSCs gating strategy: Gating was performed on CD11b^+^ cells, followed by subset classification according to Ly6G and Ly6C expression. **(B)** Representative flow cytometry plots of MDSCs differentiation. **(C)** Percentage of M-MDSCs. **(D)** Percentage of PMN-MDSCs. **(E)** Total percentage of MDSCs. **(F)** Absolute count of MDSCs (n = 5).

To confirm the effect of Emodin on the yield of differentiated MDSCs, we also assessed the absolute counts of BMC-derived MDSCs under Emodin intervention. As shown in Figure 2F, compared with the untreated group, total MDSCs counts decreased progressively in the 20 μmol/L and 40 μmol/L Emodin treatment groups (0.58 × 10^6^ ± 0.05 × 10^6^ vs. 0.49 × 10^6^ ± 0.07 × 10^6^ vs. 0.35 × 10^6^ ± 0.07 × 10^6^, *P* < 0.001), which is consistent with the previous finding that Emodin promotes MDSCs apoptosis. These results suggest that Emodin can skew BMC differentiation toward the granulocyte-like PMN-MDSCs subtype in a concentration-dependent manner.

### 3.4 Effects of Emodin on MDSCs function

Studies have shown that MDSCs can mediate immunosuppression through various pathways. To comprehensively investigate the effects of Emodin on MDSCs, this study isolated MDSCs from tumor-bearing mice via magnetic bead sorting and detected the impacts of Emodin on intracellular ROS levels, Arg-1 mean fluorescence intensity (MFI), and *Arg-1* mRNA expression in MDSCs. As shown in Figure 3, Emodin exerts divergent regulatory impacts on the immunosuppressive functions of MDSCs at different treatment concentrations. At 20 μmol/L, Emodin significantly elevated intracellular ROS levels in MDSCs (MFI: 45,769 ± 4,176 vs. 52,347 ± 4,389, *P* < 0.05) and upregulated Arg-1 expression (MFI: 1,449 ± 429 vs. 2,840 ± 709, *P* < 0.01). Specifically, Arg-1 expression was markedly increased in M-MDSCs (MFI: 1,375 ± 417 vs. 2,700 ± 719, *P* < 0.01), and a comparable rise was also detected in PMN-MDSCs (MFI: 74.42 ± 21.03 vs. 140.00 ± 30.06, *P* < 0.01). At 40 μmol/L, Emodin lowered intracellular ROS levels in MDSCs (MFI: 45,769 ± 4,176 vs. 39,367 ± 1,586, *P* < 0.05) and repressed Arg-1 expression (MFI: 1,449 ± 429 vs. 590 ± 72, *P* < 0.05). Arg-1 expression was markedly reduced in M-MDSCs (MFI: 1,375 ± 417 vs. 526 ± 86, *P* < 0.05), whereas no statistically significant differences were observed in PMN-MDSCs (MFI: 74.42 ± 21.03 vs. 63.76 ± 21.62, *P* > 0.05). Consistent with the above flow cytometry data, 20 μmol/L Emodin boosted *Arg-1* mRNA levels (1.00 ± 0.00 vs. 1.72 ± 0.45, *P* < 0.01), whereas 40 μmol/L Emodin strongly suppressed *Arg-1* transcription (1.00 ± 0.00 vs. 0.13 ± 0.01, *P* < 0.01). These findings demonstrate that Emodin triggers distinct changes in ROS and Arg-1 production within MDSCs when applied at different concentrations.

**FIGURE 3:**
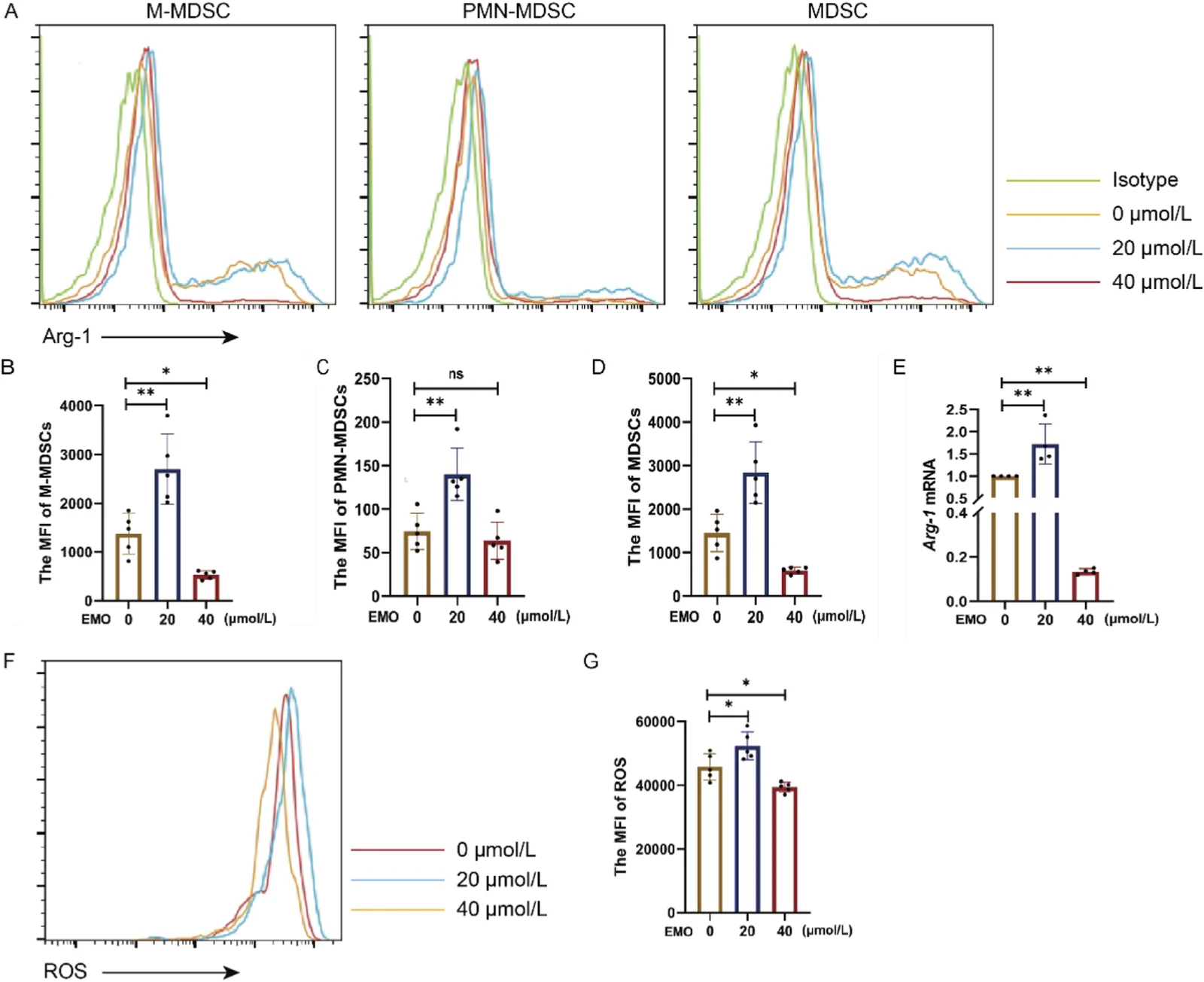
Effects of Emodin on MDSCs function. **(A)** Representative flow cytometry plots of Arg-1. **(B)** MFI of Arg-1 in M-MDSCs (n = 5). **(C)** MFI of Arg-1 in PMN-MDSCs (n = 5). **(D)** MFI of Arg-1 in MDSCs (n = 5). **(E)** Relative mRNA expression of *Arg-1* in MDSCs (n = 4). **(F)** Representative flow cytometry plots of ROS. **(G)** MFI of ROS in MDSCs (n = 5).

To further explore the regulatory effect of Emodin on MDSCs immunosuppression, MDSCs were isolated from tumor-bearing mice via magnetic bead sorting. Splenocytes were stimulated with Con A and cultured under different conditions: single culture, Emodin monotherapy, or co-culture with MDSCs with or without Emodin intervention. T cell proliferation was subsequently detected by flow cytometry. As shown in Figure 4, compared with Con A-stimulated splenocytes, the addition of MDSCs significantly reduced T cell proliferation (88.78% ± 3.95% vs. 57.20% ± 7.67%, *P* < 0.01), verifying the immunosuppressive activity of MDSCs. Meanwhile, treatment with 20 μmol/L or 40 μmol/L Emodin alone also markedly inhibited T cell proliferation in a concentration-related manner (88.78% ± 3.95% vs. 52.36% ± 12.95% vs. 28.98% ± 13.03%, *P* < 0.001). Further comparative analysis based on the MDSCs co-culture system showed that 20 μmol/L Emodin further strengthened MDSCs-mediated T cell suppression (57.20% ± 7.67% vs. 28.52% ± 11.45%, *P* < 0.05). However, 40 μmol/L Emodin did not exert additional inhibitory effects on T cell proliferation in the presence of MDSCs (28.98% ± 13.03% vs. 27.64% ± 12.11%, *P* > 0.05).

**FIGURE 4:**
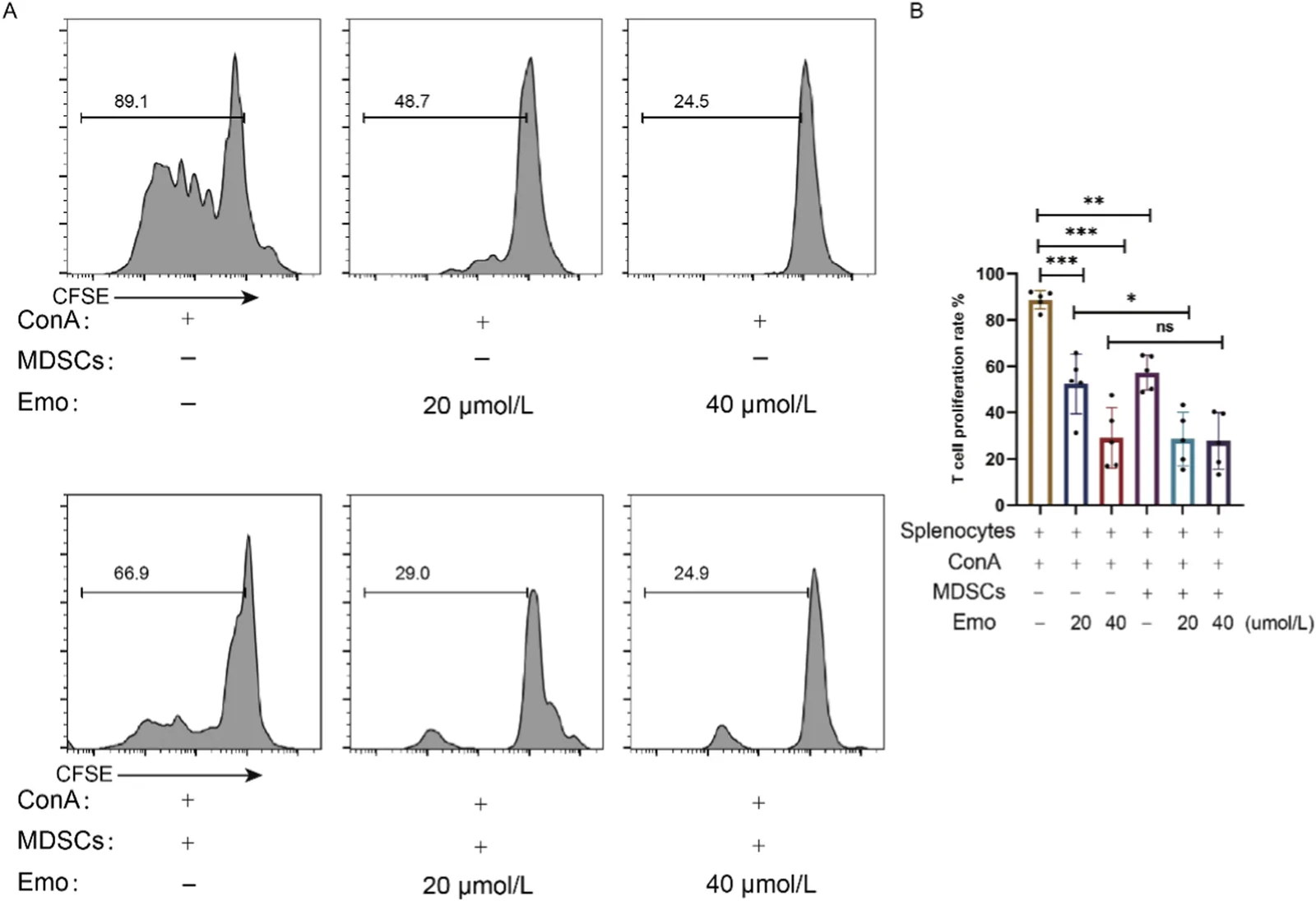
Effects of Emodin on MDSCs function. **(A)** Representative flow cytometry plots of T cell proliferation. **(B)** Percentage change in T cell proliferation (n = 5).

These results demonstrate that Emodin directly represses T cell proliferation. In addition, 20 μmol/L Emodin, but not 40 μmol/L Emodin, enhances the immunosuppressive function of MDSCs.

### 3.5 Emodin inhibits migration, proliferation, and viability of H7 cells

The malignant phenotypes including aberrant proliferation and migration of tumor cells directly contribute to unfavorable clinical prognosis. Studies have demonstrated that Emodin exerts inhibitory activities against multiple tumor cell lines, yet its functional impacts on mouse pancreatic cancer H7 cells remain poorly characterized. This study performed wound scratch assays, FCM and CCK-8 assays to evaluate the influences of different concentrations of Emodin on the migration, proliferation and viability of H7 cells. As shown in Figure 5, compared with the untreated group, all tested concentrations of Emodin significantly suppressed the migration ability of H7 cells (41.60% ± 11.63% vs. 19.60% ± 5.27% vs. 9.00% ± 1.00%, *P* < 0.001). In addition, Emodin exerted a steady concentration-dependent inhibitory effect on the proliferation (82.74% ± 12.30% vs. 73.00% ± 18.82% vs. 24.70% ± 11.13%, *P* < 0.001) and viability of H7 cells (100% ± 0.00% vs. 86.04% ± 2.55% vs. 65.38% ± 0.88%, *P* < 0.001). These results indicate that Emodin is able to suppress the malignant biological behaviors of H7 cells, including migration and proliferation, in a concentration-dependent manner.

**FIGURE 5:**
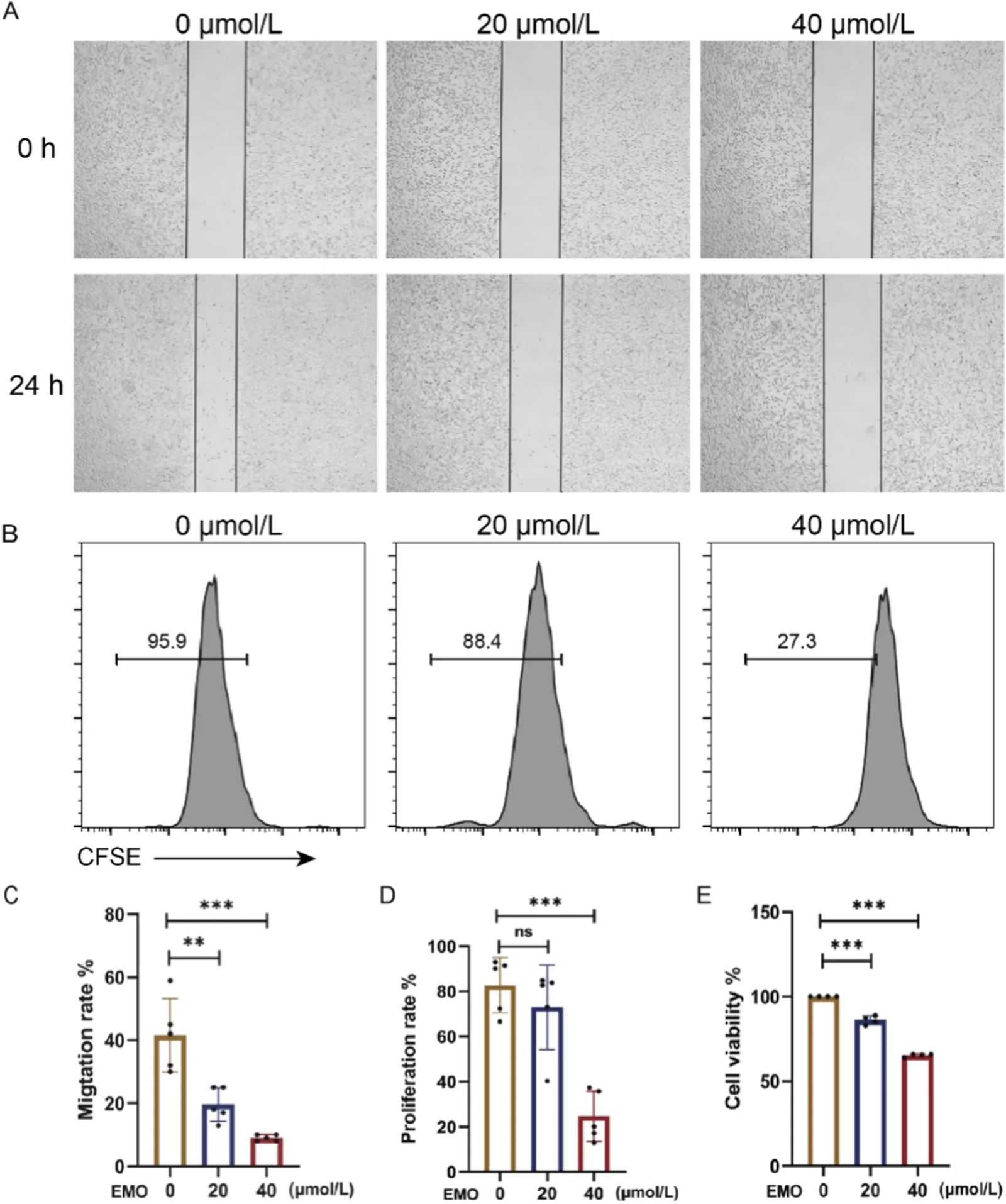
Effects of Emodin on H7 cells migration, proliferation and viability. **(A)** Representative images of wound healing assay for H7 cells. **(B)** Representative flow cytometry plots showing H7 cell proliferation. **(C)** Migration percentage of H7 cells (n = 5). **(D)** Proliferation percentage of H7 cells (n = 5). **(E)** Viability percentage of H7 cells (n = 4).

### 3.6 Emodin effectively inhibits progression of mouse pancreatic cancer

To systematically evaluate Emodin’s inhibitory effects on pancreatic cancer and its immunomodulatory effects *in vivo*, a mouse orthotopic pancreatic cancer model was established and subjected to the treatment protocol depicted in Figure 6A. During Emodin treatment, the body weight of the three mouse groups did not significantly decrease (18.28 ± 0.68 g vs. 18.24 ± 0.53 g vs. 18.54 ± 0.34 g, *P* > 0.05). *In vivo* results showed that, compared with the solvent control group, the 80 mg/kg Emodin treatment group had significantly reduced tumor weight (0.95 ± 0.07 g vs. 0.58 ± 0.11 g, *P* < 0.001), while the 40 mg/kg Emodin treatment group showed no significant change in tumor weight (0.95 ± 0.07 g vs. 0.92 ± 0.08 g, *P* > 0.05). These results demonstrate that high-dose Emodin exhibits prominent anti-pancreatic cancer activity *in vivo* and possesses favorable biosafety.

**FIGURE 6:**
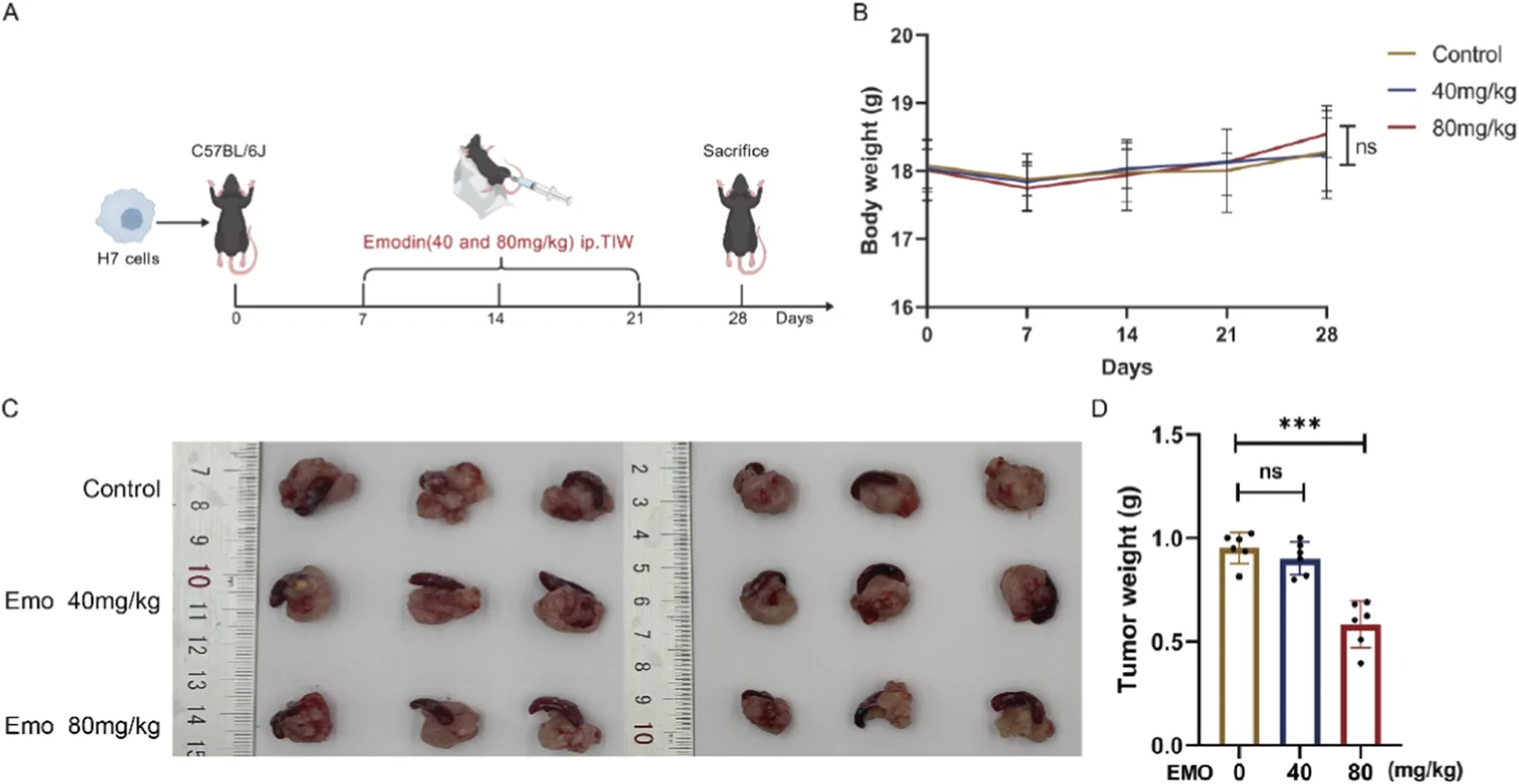
Establishment of mouse orthotopic pancreatic cancer model and effects of Emodin treatment. **(A)** Construction and treatment protocol of the mouse orthotopic pancreatic cancer model. **(B)** Body weight changes in each group during treatment. **(C)** Tumor images of each group in the orthotopic pancreatic cancer model. **(D)** Tumor weights of each group (n = 6).

### 3.7 Effects of Emodin on splenic MDSCs and Treg in mouse pancreatic cancer

In pancreatic cancer research, the spleen acts as a vital peripheral immune organ and an important indicator for assessing systemic immune homeostasis and tumor-associated immune responses. To explore how Emodin shapes systemic immunity in mouse orthotopic pancreatic cancer models, we analyzed shifts in splenic MDSCs frequencies. As shown in Figure 7B, administration of 40 mg/kg or 80 mg/kg Emodin markedly lowered total splenic MDSCs frequencies relative to the solvent control group (50.11% ± 3.86% vs. 45.63% ± 2.11% vs. 41.30% ± 4.65%, *P* < 0.01). Emodin also remodeled MDSCs subpopulations: it decreased M-MDSCs percentages (49.38% ± 3.88% vs. 44.90% ± 1.96% vs. 39.85% ± 1.23%, *P* < 0.01) while raising PMN-MDSCs percentages (0.73% ± 0.12% vs. 0.73% ± 0.24% vs. 1.45% ± 0.64%, *P* < 0.05).

**FIGURE 7:**
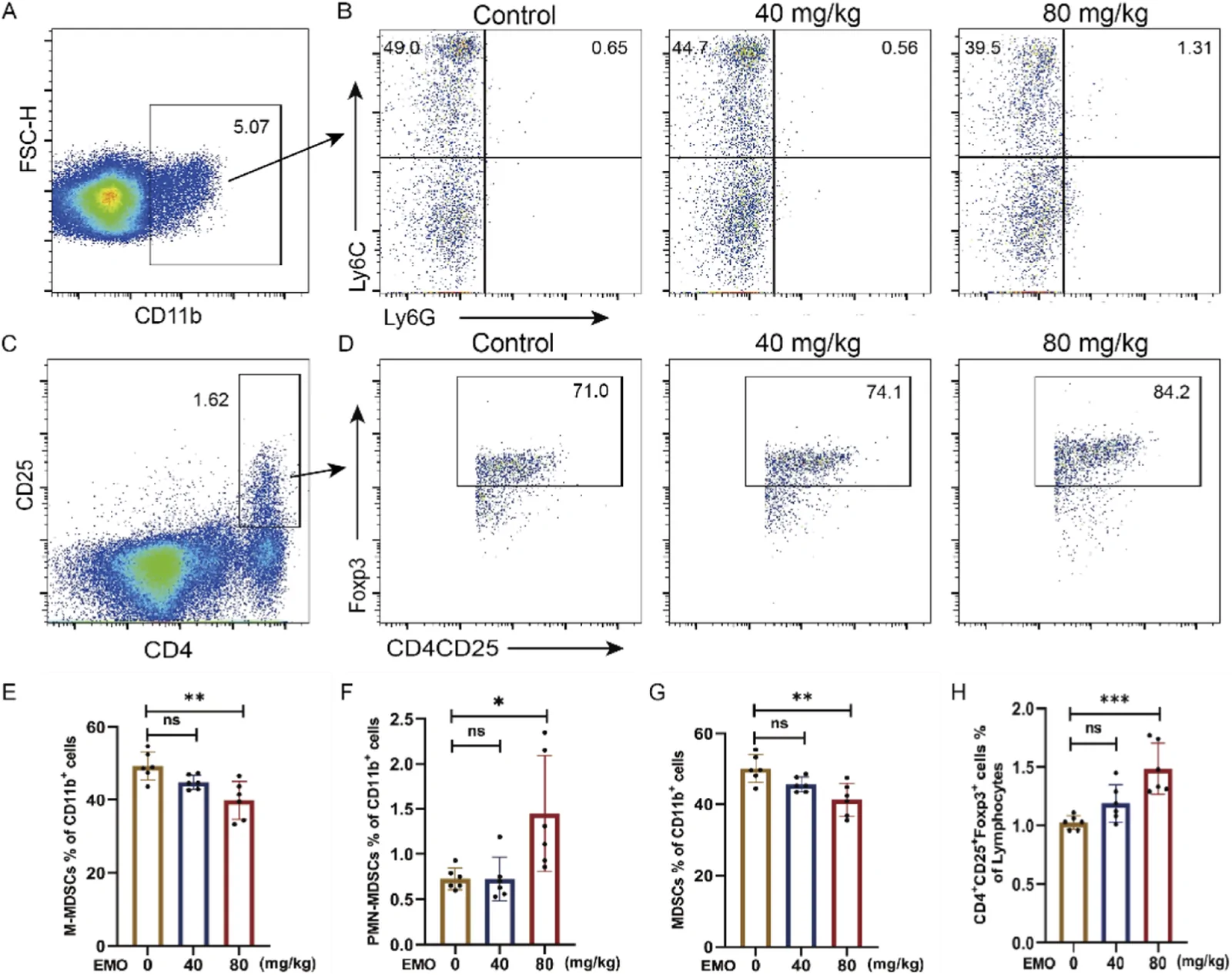
Proportional changes in spleen MDSCs and Treg after Emodin treatment. **(A)** Splenic MDSCs gating strategy: CD11b^+^ cells were gated, and subsets were further distinguished based on Ly6G and Ly6C expression. **(B)** Representative flow cytometry plots of splenic MDSCs. **(C)** Splenic Treg gating strategy: CD4^+^CD25^+^ cells were gated, and Treg were identified according to Foxp3 expression levels. **(D)** Representative flow cytometry plots of splenic Treg **(E)** Percentage of M-MDSCs. **(F)** Percentage of PMN-MDSCs. **(G)** Percentage of MDSCs. **(H)** Percentage of Treg (n = 6).

Splenic Treg, core mediators of systemic immunosuppression, were further quantified. As illustrated in Figure 7D, both 40 mg/kg and 80 mg/kg Emodin treatments elevated splenic Treg frequencies compared with the solvent control group (1.03% ± 0.06% vs. 1.20% ± 0.16% vs. 1.50% ± 0.22%, *P* < 0.001).

These results reveal that Emodin reduces total splenic MDSCs levels with distinct effects across tested doses and remodels their subset balance, with fewer M-MDSCs and more PMN-MDSCs observed. In addition, Emodin intervention upregulates splenic Treg frequencies, suggesting its modulatory role in systemic immunity.

### 3.8 Effects of Emodin on splenic T cells and their subsets in mouse pancreatic cancer

In systemic immune responses to pancreatic cancer, the number and composition of splenic T cells are important indicators of antitumor immune function. CD4^+^ T cells participate in immune regulation via antigen presentation, helper function and cytokine secretion, whereas CD8^+^ T cells specifically recognize antigen-MHC I complexes to directly eliminate tumor cells. To investigate Emodin’s influence on splenic T cell populations, we detected the proportions of CD3^+^ T cells and their subsets following Emodin administration. As shown in Figure 8, both 40 mg/kg and 80 mg/kg Emodin groups exhibited lower splenic CD3^+^ T cell proportions compared with the solvent control group (29.87% ± 12.26% vs. 21.95% ± 8.55% vs. 16.42% ± 3.10%, *P* < 0.05). Subset analysis showed a remarkable drop in CD8^+^ T cell percentage (34.13% ± 5.18% vs. 31.30% ± 6.03% vs. 24.70% ± 7.03%, *P* < 0.05), whereas CD4^+^ T cell proportions had no statistical differences among groups (43.12% ± 6.00% vs. 46.20% ± 4.32% vs. 48.65% ± 7.87%, *P* > 0.05).

**FIGURE 8:**
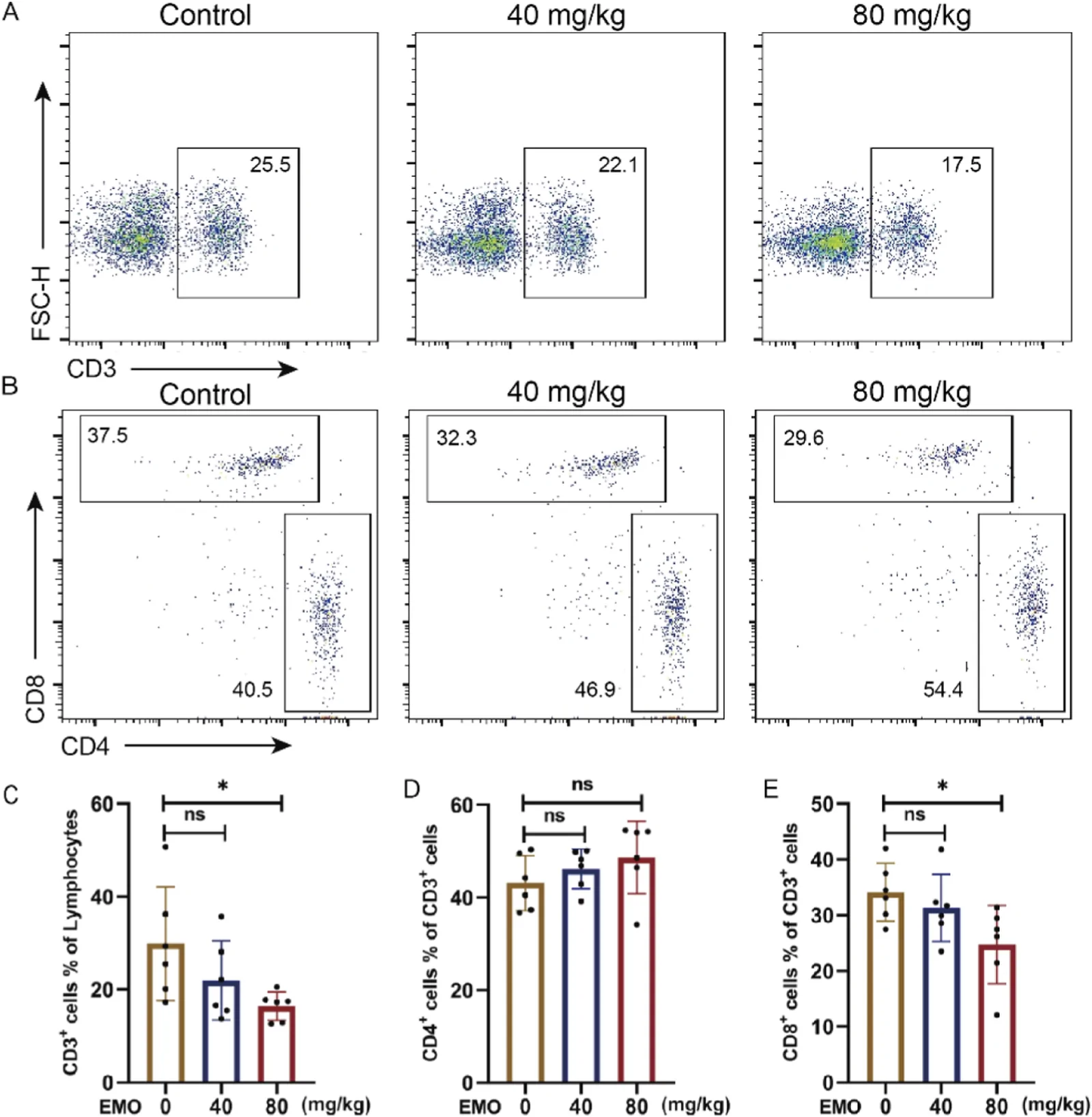
Proportional changes in splenic T cells and their subsets after Emodin treatment. **(A)** Representative flow cytometry plots of CD3^+^ T cells in mouse splenic lymphocytes. **(B)** Representative flow cytometry plots of CD4^+^ T and CD8^+^ T cells in mouse splenic lymphocytes. **(C)** Percentage of CD3^+^ T cells in mouse splenic lymphocytes. **(D)** Percentage of CD3^+^CD4^+^ T cells in splenic lymphocytes. **(E)** Percentage of CD3^+^CD8^+^ T cells in splenic lymphocytes (n = 6).

These findings suggest Emodin treatment causes a general decline in splenic T cell proportions, especially CD8^+^ T cells.

### 3.9 Effects of Emodin on MDSCs and Treg in the tumor immune microenvironment of mouse pancreatic cancer

The dual immunosuppression mediated by MDSCs and Treg in the tumor immune microenvironment of pancreatic cancer plays a pivotal role in tumor immune evasion and serves as a target for immune combination therapies. This study evaluated the impact of Emodin on tumor-infiltrating MDSCs and Treg in tumor-bearing mice. As shown in Figure 9B, treatment with Emodin did not significantly alter the overall proportion of MDSCs in the tumor immune microenvironment compared with the solvent control group (26.23% ± 5.05% vs. 24.47% ± 3.32% vs. 27.49% ± 4.40%, *P* > 0.05). However, significant changes were observed in the subsets of MDSCs, with M-MDSCs proportion decreasing (18.08% ± 5.22% vs. 13.83% ± 3.50% vs. 8.57% ± 4.60%, *P* < 0.01) and PMN-MDSCs proportion increasing (8.15% ± 4.85% vs. 10.64% ± 3.34% vs. 18.92% ± 7.14%, *P* < 0.01). The infiltration level of Treg in tumor tissues was also assessed and is shown in Figure 9D. Emodin treatment significantly increased the proportion of Treg in tumor tissues at both 40 mg/kg and 80 mg/kg doses compared with the solvent control group (0.26% ± 0.16% vs. 0.30% ± 0.13% vs. 0.49% ± 0.17%, *P* < 0.05).

**FIGURE 9:**
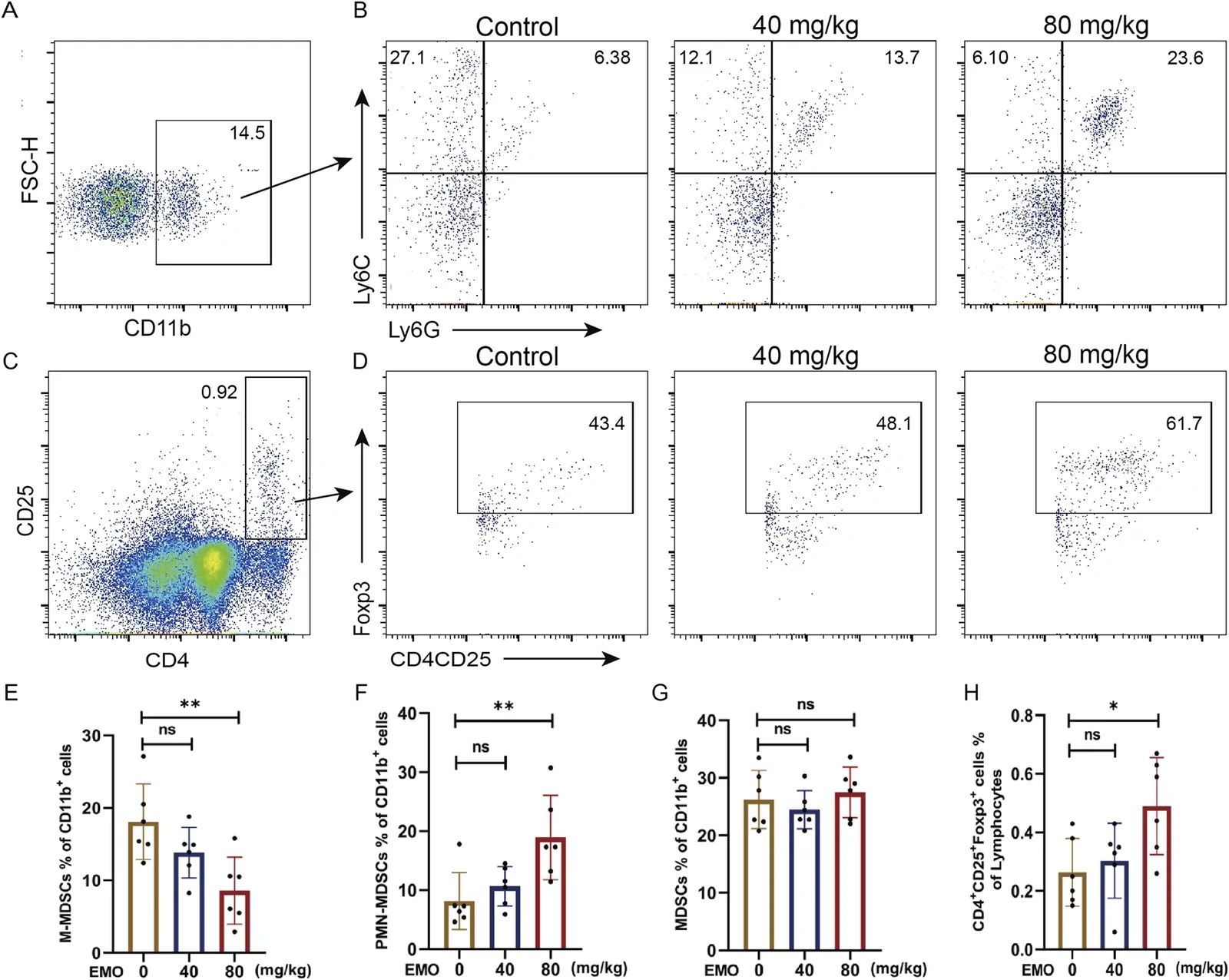
Changes in proportions of MDSCs and Treg in the tumor microenvironment of mice after Emodin treatment. **(A)** Tumor MDSCs gating strategy: CD11b^+^ cells were gated, and subsets were further distinguished based on Ly6G and Ly6C expression. **(B)** Representative flow cytometry plots of MDSCs in the tumor microenvironment. **(C)** Tumor Treg gating strategy: CD4^+^CD25^+^ cells were gated, and Treg were identified according to Foxp3 expression levels. **(D)** Representative flow cytometry plots of Treg in the tumor microenvironment. **(E)** Percentage of M-MDSCs. **(F)** Percentage of PMN-MDSCs. **(G)** Percentage of MDSCs. **(H)** Percentage of Treg (n = 6).

These results indicate that while Emodin modifies the subset composition of tumor-resident MDSCs, it does not significantly affect their overall proportion. Additionally, Emodin treatment enhances Treg infiltration within the tumor microenvironment, potentially contributing to local immune modulation.

### 3.10 Effects of Emodin on T cells and their subsets in the tumor immune microenvironment of mouse pancreatic cancer

Pancreatic cancer is characterized by low immunogenicity due to insufficient effector T cell infiltration and dysfunction, including the reduction and exhaustion of CD8^+^ T cells, which compromises their tumor-killing ability. CD4^+^ T cells are prone to differentiating into immunosuppressive Treg, contributing to poor outcomes with immunotherapy. This study revealed that Emodin did not decrease the levels of MDSCs in the tumor immune microenvironment but significantly increased Treg infiltration. To assess its impact on tumor-infiltrating CD3^+^ T cells, analysis of their proportion and distribution was conducted. As shown in Figure 10, Emodin treatment resulted in a significant reduction in the proportion of CD3^+^ T cells in the tumor tissues compared with the solvent control group (45.52% ± 6.48% vs. 43.22% ± 4.30% vs. 35.63% ± 1.93%, *P* < 0.01). Subgroup analysis showed a marked decrease in CD8^+^ T cell proportion (27.65% ± 5.05% vs. 24.63% ± 3.88% vs. 16.06% ± 5.40%, *P* < 0.01), while CD4^+^ T cell proportions did not exhibit a statistically significant change (45.47% ± 2.59% vs. 49.60% ± 5.59% vs. 43.55% ± 4.35%, *P* > 0.05).

**FIGURE 10:**
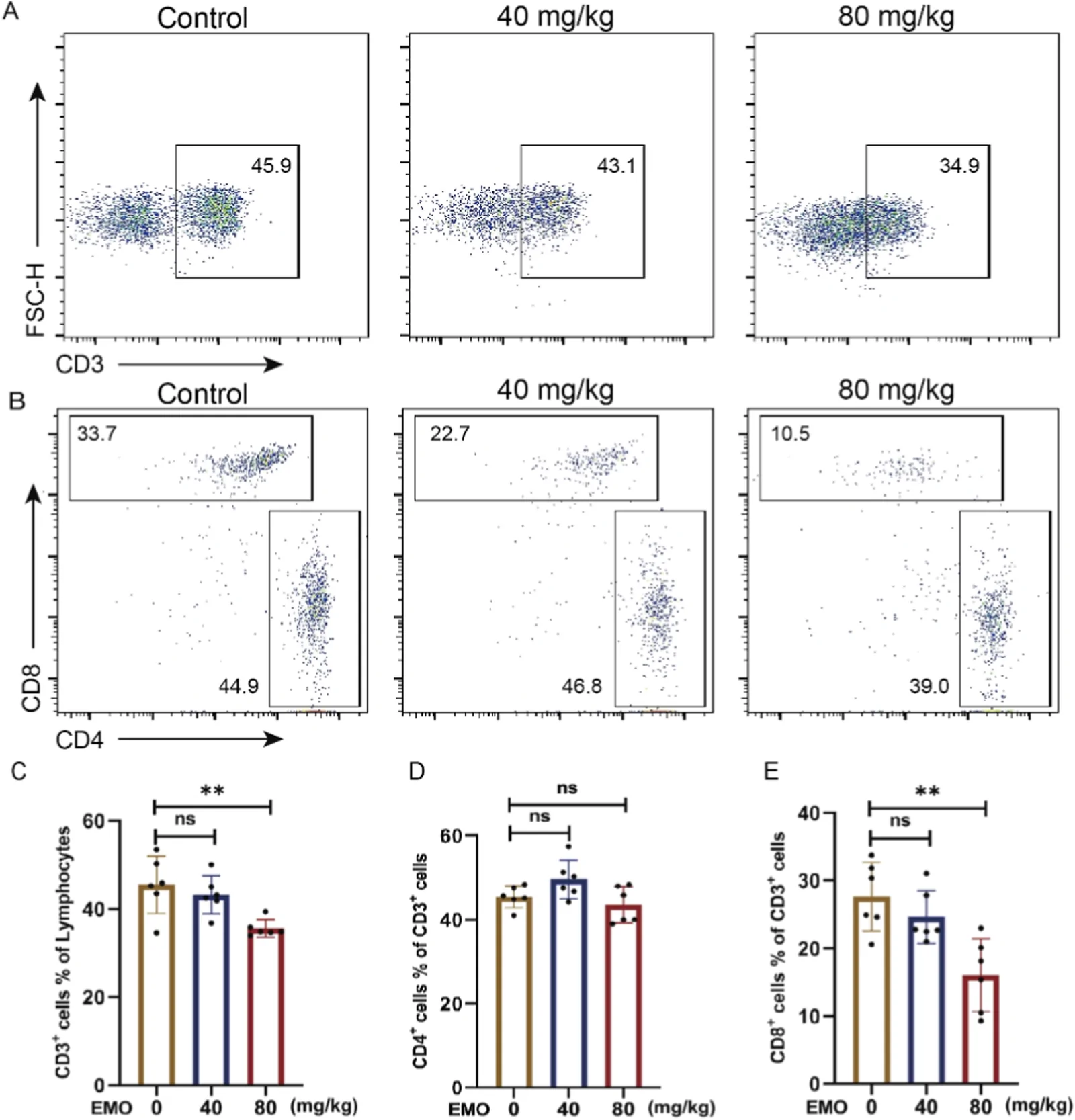
Changes in proportions of T cells and their subsets in the tumor immune microenvironment of mice after Emodin treatment. **(A)** Representative flow cytometry plots of CD3^+^ T cells in mouse tumor-infiltrating lymphocytes. **(B)** Representative flow cytometry plots of CD4^+^ T and CD8^+^ T cells in mouse tumor-infiltrating lymphocytes. **(C)** Percentage of CD3^+^ T cells in mouse tumor-infiltrating lymphocytes. **(D)** Percentage of CD3^+^CD4^+^ T cells in mouse tumor-infiltrating lymphocytes. **(E)** Percentage of CD3^+^CD8^+^ T cells in mouse tumor-infiltrating lymphocytes (n = 6).

These results suggest that Emodin suppresses the infiltration of intratumoral CD3^+^ T cells, particularly CD8^+^ T cells, potentially leading to a weakened anti-tumor immune response.

### 3.11 Molecular mechanism of Emodin in regulating MDSCs

In the tumor immune microenvironment, cytokines such as GM-CSF and IL-6 can inhibit the apoptosis of MDSCs by activating the STAT3 pathway. This pathway can upregulate the expression of anti-apoptotic proteins such as Bcl-2. As shown in Figure 11A, compared with the control group, treatment with 20 μmol/L or 40 μmol/L Emodin markedly decreased STAT3 phosphorylation in MDSCs (0.98 ± 0.14AU vs. 0.82 ± 0.12 AU vs. 0.62 ± 0.09 AU, *P* < 0.01).

**FIGURE 11:**
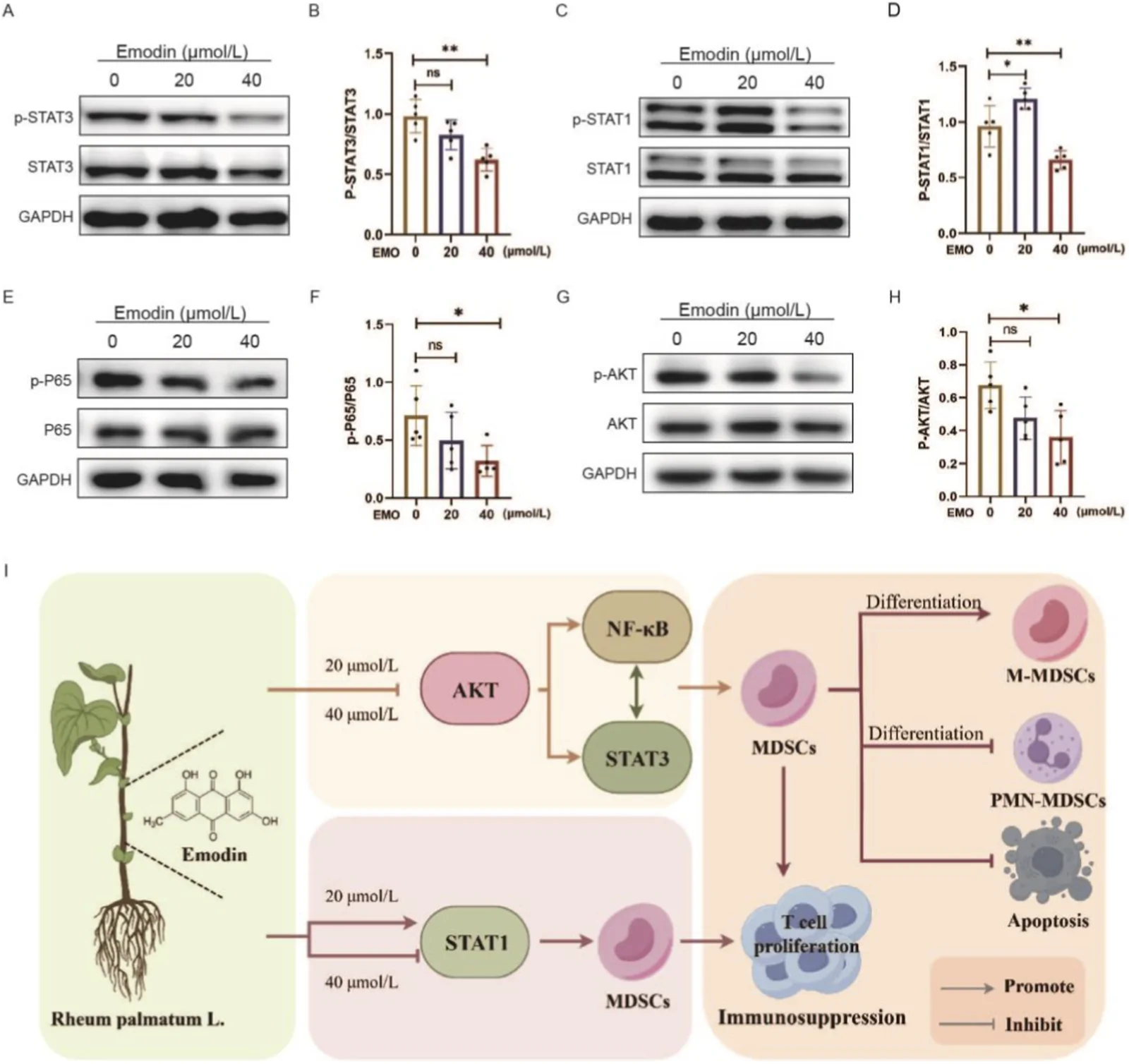
Effects of Emodin on AKT/NF-κB/STAT3/STAT1 signaling pathways in MDSCs. **(A)** Representative Western blot images of STAT3 signaling pathway proteins in MDSCs. **(B)** Quantitative analysis of STAT3 phosphorylation levels in MDSCs. **(C)** Representative Western blot images of STAT1 signaling pathway proteins in MDSCs. **(D)** Quantitative analysis of STAT1 phosphorylation levels in MDSCs. **(E)** Representative Western blot images of NF-κB p65 signaling pathway proteins in MDSCs. **(F)** Quantitative analysis of NF-κB p65 phosphorylation levels in MDSCs. **(G)** Representative Western blot images of AKT signaling pathway proteins in MDSCs. **(H)** Quantitative analysis of AKT phosphorylation levels in MDSCs. **(I)** Schematic diagram illustrating the regulatory mechanism of the AKT/NF-κB/STAT3/STAT1 signaling axis (n = 5).

The STAT1 signaling cascade serves as another critical axis governing the biological functions of MDSCs. As shown in Figure 11C, compared with the control group, 20 μmol/L Emodin upregulated the phosphorylation level of STAT1 in MDSCs, whereas 40 μmol/L Emodin significantly suppressed STAT1 phosphorylation (0.96 ± 0.19AU vs. 1.21 ± 0.10AU vs. 0.66 ± 0.08AU, *P* < 0.01).

Apart from STAT family pathways, NF-κB and AKT are upstream signaling molecules that mediate MDSCs survival, proliferation and immunosuppressive functions; extensive signal crosstalk exists between these molecules and STAT pathways. To further clarify the multi-target molecular mechanism by which Emodin regulates MDSCs, we also detected the phosphorylation levels of NF-κB p65 and AKT. As shown in Figure 11E, Emodin significantly suppressed NF-κB p65 phosphorylation in a dose-dependent manner (0.71 ± 0.26AU vs. 0.50 ± 0.24AU vs. 0.32 ± 0.13AU, *P* < 0.05). Consistent with this, Figure 11G showed that Emodin markedly decreased AKT phosphorylation in MDSCs (0.68 ± 0.14AU vs. 0.48 ± 0.13AU vs. 0.37 ± 0.16AU, *P* < 0.05).

These results indicate that Emodin modulates MDSCs apoptosis, differentiation and function via the AKT/NF-κB/STAT3/STAT1 signaling pathways (Figure 11).

## 4 Discussion

The pancreatic tumor immune microenvironment is heavily infiltrated by immunosuppressive populations including MDSCs and Treg, which drive tumor immune evasion via the secretion of inhibitory cytokines such as IL-10, TGF-β and VEGF (Yu et al., 2025). As a heterogeneous subset of immune-suppressive myeloid cells, MDSCs accelerate tumor progression and metastasis by boosting tumor cell proliferation and facilitating epithelial–mesenchymal transition. They also generate suppressive mediators including ROS and Arg-1 to block T cell activation and expansion, ultimately sustaining tumor cell survival (Abdalsalam et al., 2024). Sustained MDSCs’ viability and their resistance to apoptotic stimuli are critical for maintaining persistent immunosuppression within the tumor microenvironment. Gemcitabine exerts direct cytotoxic effects on tumor cells while diminishing MDSCs’ abundance and functional activity, consequently amplifying T cell-dependent antitumor immunity (Siegel et al., 2023). Likewise, in murine tumor models, 5-FU suppresses tumor growth through selective induction of MDSCs’ apoptosis (Kim et al., 2021). In the present study, we verified that Emodin triggers MDSCs’ apoptosis (Figure 1). This effect reduces the aberrant accumulation of MDSCs in peripheral immune organs (Figure 7), and relieves MDSCs-dominant immunosuppression. Notably, although Emodin robustly induces MDSCs’ apoptosis and reduces MDSCs counts both *in vitro* and in the spleen, total intratumoral MDSC numbers show no obvious decline (Figure 9). Two potential mechanisms may account for this discrepancy. First, tumor-mediated recruitment: Tumor tissue continuously recruits circulating MDSCs populations from peripheral reservoirs such as the spleen, offsetting the local apoptotic effect triggered by Emodin. Second, restricted drug penetration: Pancreatic cancer features a dense extracellular matrix with sparse vasculature that acts as a physical barrier (Dauer et al., 2017). Such stromal architecture impedes efficient intratumoral penetration of Emodin, leading to insufficient intratumoral drug concentrations to elicit robust MDSCs’ apoptosis.

GM-CSF serves as a crucial growth factor in the tumor microenvironment that governs the generation, survival, and immunosuppressive capacity of MDSCs. It modulates MDSCs’ biological behaviors via multiple signaling cascades, including AKT, STAT3, and NF-κB. Specifically, GM-CSF-mediated STAT3 activation promotes MDSCs’ proliferation, survival, and immunosuppressive activity. Moreover, NF-κB signaling activation drives MDSCs’ differentiation and stimulates the production of immunosuppressive mediators, such as ROS, which are essential for MDSCs-mediated inhibition of T-cell immune responses (Wang et al., 2025). AKT lies upstream of the NF-κB and STAT3 signaling pathways. There is extensive crosstalk between NF-κB and STAT3, which together constitute the STAT3-NF-κB signaling axis (Grivennikov and Karin, 2010). Activated STAT3 upregulates anti-apoptotic proteins such as Bcl-2, suppresses MDSCs’ apoptosis and facilitates their massive accumulation (RéBé et al., 2013). Previous research has confirmed that Emodin regulates the AKT/NF-κB signaling pathway (Chen et al., 2023; Gao et al., 2024). This study demonstrates that Emodin simultaneously downregulates phosphorylation levels of AKT, NF-κB and STAT3 in MDSCs (Figures 11A,B,E–H). Simultaneously, Emodin can promote MDSCs’ apoptosis (Figure 1) and modulate the production of ROS and Arg-1 in MDSCs under 20 μmol/L treatment (Figure 3); it not only directly suppresses T-cell proliferation but also synergistically amplifies the immunosuppressive potency of MDSCs at 20 μmol/L (Figure 4). Collectively, these data indicate that Emodin impairs MDSCs’ survival capacity at multiple layers by suppressing the AKT/NF-κB/STAT3 signaling cascade. It cooperatively initiates MDSCs’ apoptosis via STAT3 inhibition while exerting concentration-dependent enhancement on MDSCs’ immunosuppressive function.

Research has found that Emodin can inhibit M1 macrophage polarization by inhibiting the NF-κB signaling pathway (Iwanowycz et al., 2016). This study reveals that Emodin drives directional differentiation of MDSCs toward PMN-MDSCs, accompanied by a declined fraction of M-MDSCs (Figure 2). This shift can be detected in both the spleen and tumor tissue, characterized by reduced M-MDSCs and elevated proportions of PMN-MDSCs (Figures 7B, 9B). Prior research has validated that STAT3 knockout or pharmacological inhibition reshapes the developmental trajectory of myeloid cells (RéBé et al., 2013). The results of this study show that Emodin can inhibit the phosphorylation level of STAT3 in MDSCs (Figure 4A). Therefore, it can be speculated that the reduction in STAT3 activity removes its “blockage” effect on myeloid cell differentiation, which may be the upstream molecular mechanism leading to the decrease in the total number of MDSCs and the alteration of MDSCs subset distribution. Although reducing the overall number of MDSCs is usually regarded as a beneficial therapeutic strategy in anti-tumor immunotherapy, the specific differentiation of MDSCs induced by Emodin towards PMN-MDSCs may be a “double-edged sword”. Previous studies have indicated that PMN-MDSCs are associated with tumor invasion and metastasis. In the 4T1 breast cancer model, high levels of PMN-MDSCs were found to promote tumor bone metastasis (Yang et al., 2008). Inhibiting PMN-MDSCs differentiation in the bone marrow using plant-derived polyacetylenes significantly suppressed tumor metastasis (Wei et al., 2016). Accordingly, Emodin’s promotion of PMN-MDSCs differentiation might pose a risk for tumor metastasis. Further exploration of the related mechanisms is warranted.

At the functional level, M-MDSCs use iNOS to suppress T cell responses in both antigen-specific and non-antigen-specific manners, while PMN-MDSCs utilize ROS to inhibit immune responses in an antigen-specific manner, inducing antigen-specific T cell tolerance (Gabrilovich, 2017). Both types can exert immunosuppressive effects through Arg-1 (Shen et al., 2022). The activation of STAT1 is associated with the enhancement of the immunosuppressive function of MDSCs (Poe et al., 2013). The results of this study show that Emodin can directionally drive MDSCs differentiation towards the granulocyte-like PMN-MDSCs (Figure 2). Furthermore, this study observes that Emodin exerts opposite effects on STAT1 phosphorylation at different concentrations (Figure 11C). This provides a molecular mechanism for explaining its opposite effects on the release of immune-inhibitory molecules ROS and Arg-1 by MDSCs at different concentrations. At the low concentration of 20 μmol/L, Emodin upregulates STAT1 phosphorylation in MDSCs. This precisely explains why 20 μmol/L Emodin significantly increases the expression of ROS and Arg-1 in MDSCs, and synergistically enhances the inhibitory effect of MDSCs on T cell proliferation. However, at a high concentration of 40 μmol/L, this trend reverses, and STAT1 phosphorylation is markedly suppressed. The reduction in STAT1 activity attenuates the immunosuppressive function of MDSCs, manifested as decreased expression of ROS and Arg-1, rendering MDSCs incapable of synergistically inhibiting T cell proliferation. This “low stimulation and high inhibition” phenomenon is known in pharmacology as hormesis, where compounds produce stimulating effects at low doses and inhibitory effects at high doses. Three plausible mechanisms may account for this phenomenon. First, target switching: At low concentrations, Emodin preferentially targets upstream kinases of the STAT1 cascade (e.g., JAK) to moderately activate STAT1 signaling; at high concentrations, it either directly inhibits STAT1 protein or activates uncharacterized phosphatases to trigger STAT1 dephosphorylation. Second, feedback regulation: Mild STAT1 activation induced by low-dose Emodin initiates intracellular negative feedback loops, yet the robust intervention of high-concentration Emodin overrides such compensatory feedback and ultimately suppresses STAT1 signaling. Third, cellular stress response: High-dose Emodin acts as a potent cellular stressor, activating multiple signaling cascades that antagonize STAT1 and indirectly dampen STAT1 phosphorylation.

Beyond its immunomodulatory effects, Emodin also exhibits broad antitumor potency against multiple malignant cell lines. Emodin restricts the proliferation and invasion of colorectal cancer cells via blockade of VEGFR2 (Dai et al., 2019). It also represses human breast cancer cell proliferation by activating the aryl hydrocarbon receptor (Zhang et al., 2020). This study demonstrates that Emodin markedly impairs the viability, proliferation, and migration of the pancreatic cancer H7 cell line (Figure 5). In orthotopic pancreatic tumor-bearing mice, 80 mg/kg Emodin markedly slows tumor progression. By contrast, 40 mg/kg Emodin fails to yield obvious therapeutic benefits *in vivo* (Figure 6), a phenomenon likely driven by augmented immunosuppressive function of MDSCs upon low-dose Emodin exposure. However, despite Emodin’s potent direct antitumor activity, its capacity to reverse the overall immunosuppressive state of the tumor immune microenvironment remains limited. Additionally, previous studies have reported that Emodin induces Treg generation and suppresses mouse skin allograft rejection (Cao et al., 2022). This study observes a marked expansion of Treg in both the spleen and tumor immune microenvironment after Emodin intervention (Figures 7D, 10D), accompanied by a remarkable reduction in antitumor CD8^+^ T cells (Figures 8B, 11B). Treg enrichment is closely correlated with PMN-MDSCs expansion and remodeling of the immune microenvironment induced by low-dose (20 μmol/L) Emodin, which aligns with the observation that low-dose (20 μmol/L) Emodin strengthens the immunosuppressive function of MDSCs. Emodin’s intrinsic suppression of T cell proliferation further facilitates tumor immune evasion. Collectively, although Emodin modulates MDSCs biological behaviors, it concurrently reinforces the immunosuppressive dominance of Treg.

In summary, Emodin effectively suppresses malignant phenotypes of pancreatic cancer cells, including proliferation, invasion and migration. It modulates the AKT/NF-κB/STAT3/STAT1 signaling cascade, induces MDSCs apoptosis, and selectively remodels MDSCs subset distribution. Notably, Emodin exhibits distinct dose-dependent regulatory effects on the immunosuppressive capacity of MDSCs: low-dose Emodin augments MDSCs-mediated immune suppression, while high-dose Emodin attenuates these inhibitory activities. Moreover, Emodin increases the frequency of Treg and reduces the abundance of anti-tumor CD8^+^ T cells. These immunomodulatory shifts prevent complete reprogramming of the immunosuppressive tumor microenvironment, representing a major limitation of Emodin monotherapy.

This study has several limitations. First, only a single pancreatic cancer cell line and female mice were utilized in the experiments, which limits the generalizability of the findings. Second, the dense stromal barrier of pancreatic cancer impedes the intratumoral penetration of Emodin. To overcome these shortcomings, future studies will optimize nano-targeted delivery systems to disrupt the stromal barrier, perform multi-model validations, and explore Emodin-based combination therapeutic strategies. These prospective improvements are expected to remodel the immunosuppressive tumor microenvironment, overcome the limitations of monotherapy, and further enhance the clinical translational potential of Emodin in pancreatic cancer treatment.

## Data Availability

The original contributions presented in the study are included in the article/Supplementary Material, further inquiries can be directed to the corresponding authors.

## Supplementary material

SUPPLEMENTARY FIGURE S1 Dose-response fitting of Emodin cytotoxicity.

SUPPLEMENTARY FIGURE S2 Toxicity detection of Emodin in mice **(A)** Representative HE staining images of liver tissues. **(B)** Representative HE staining images of kidney tissues. **(C)** Serum levels of ALT. **(D)** Serum levels of AST. **(E)** Serum levels of CRE. **(F)** Serum levels of BUN. (n=5).
