# Correction: Effect and mechanism of Emodin on mouse myeloid-derived suppressor cells

**DOI:** 10.3389/fphar.2026.2001607

**Published:** 2026-10-12

**Authors:** Frontiers Production Office

**Affiliations:** Frontiers Media SA, Lausanne, Switzerland

**Keywords:** AKT/NF-κB/STAT3/STAT1, Emodin, myeloid-derived suppressor cells, pancreatic cancer, tumor immune microenvironment

Figures 4–11 were in the wrong order in the published article. The originally published Figure 11 should have been Figure 4. Consequently, all other figures have been renumbered, and the order has now been corrected. Additionally, while the correct Figure 4 was cited correctly in section “3.4 Effects of Emodin on MDSCs function”, subsequent figure citations of Figures 5–11 were incorrect (for example, in section “3.5 Emodin inhibits migration, proliferation, and viability of H7 cells”, the text should cite Figure 5, not Figure 4; in section “3.6 Emodin effectively inhibits progression of mouse pancreatic cancer”, the text should cite Figure 6A, not Figure 5A, and so on.)

The original version of this article has been updated.

